# Research on Concrete Columns Reinforced with New Developed High-Strength Steel under Eccentric Loading

**DOI:** 10.3390/ma12132139

**Published:** 2019-07-03

**Authors:** Yonghui Hou, Shuangyin Cao, Xiangyong Ni, Yizhu Li

**Affiliations:** 1 Key Laboratory of Concrete and Prestressed Concrete Structures of Ministry of Education, Southeast University, Nanjing 211189, China; 2 School of Civil Engineering, Southeast University, Nanjing 211189, China

**Keywords:** concrete columns, eccentric loading, high-strength steel, ductility, bearing capacity

## Abstract

The use of new developed high-strength steel in concrete members can reduce steel bar congestion and construction costs. This research aims to study the behavior of concrete columns reinforced with new developed high-strength steel under eccentric loading. Ten reinforced concrete columns were fabricated and tested. The test variables were the transverse reinforcement amount and yield strength, eccentricity, and longitudinal reinforcement yield strength. The failure patterns were compression and tensile failure for columns subjected to small eccentricity and large eccentricity, respectively. The same level of post-peak deformability and ductility could only be obtained with a lower amount of transverse reinforcement when high-strength transverse reinforcements were used in columns subjected to small eccentricity. The high-strength longitudinal reinforcement improved the bearing capacity and post-peak deformability of the concrete columns. Furthermore, three different equivalent rectangular stress block (ERSB) parameters for predicting the bearing capacity of columns with high-strength steel are discussed based on test and simulated results. It is concluded that the China Code GB 50010-2010 overestimates the bearing capacity of columns with high-strength steel, whereas the bearing capacities computed using the America Code ACI 318-14 and Canada Code CSA A23.3-04 agree well with the test results.

## 1. Introduction

**Figure 1:**
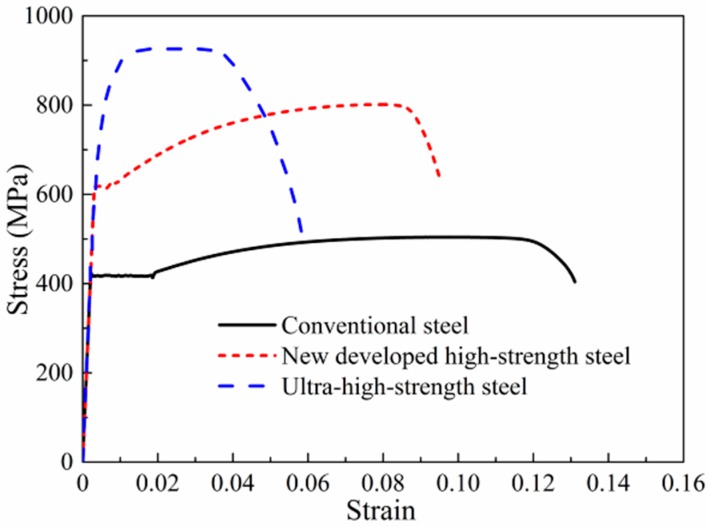
Stress–strain relationships of different reinforcing bars.

Over the last decade, the use of high-strength steel bars in the construction industry has prompted extensive research in this area. High-strength steel bars have the advantage of lowering reinforcement congestion and construction costs, especially in high-rise and special buildings. The use of high-strength steel as longitudinal reinforcement can enhance concrete members’ load capacity; moreover, its use for stirrups may decrease the transverse reinforcement amount required to ease concrete placement. In recent years, the continuous development of steel smelting technology has produced new high-strength steel (for example, Grade 100 in USA, Grade 600 in Korea, and HRB600 in China). The new developed high-strength steel has a linear pre-yield behavior, obvious yield plateau and comparatively good ductility, while ultra-high-strength reinforcing bars have a high yield strength, but no yield plateau and poor ductility. The typical stress–strain relationships of different reinforcing bars are presented in Figure 1. The yield plateau of new developed high-strength steel is much shorter than that of conventional steel, and the rupture elongation of new developed high-strength steel is approximately 70% that of conventional steel. Therefore, the new developed high-strength steel (with its altered mechanical properties) has an obvious effect on the performance of concrete members.

There have been many investigations on the performance of high-strength steel (including high-strength longitudinal reinforcement and transverse reinforcement) used in concrete beams [1,2,3], beam–column joints [4,5,6], and walls [7,8]. As a result, the use of high-strength steel has become widespread in concrete structural applications. However, limited research has been carried out to investigate the eccentric compressive behavior of concrete columns reinforced with high-strength steel, although many scholars [9,10,11,12,13,14] have conducted studies on high-strength steel used in concrete columns.

Many researchers [15,16,17] have carried out axial compression tests on concrete columns confined by high-strength transverse reinforcements and demonstrated that the use of high-strength stirrups can improve the ductility behavior of columns and results in reduced steel congestion during construction. Paultre et al. [18] and Xiao et al. [19] performed experimental studies on concrete columns confined with high-strength transverse reinforcements under reversed cyclic loading and mentioned that the seismic behavior of the columns increased. Moreover, high-strength transverse reinforcement can effectively confine concrete while reducing the transverse reinforcement amount. Rautenberg et al. [9,10] studied eight concrete columns reinforced with high-strength longitudinal reinforcements under displacement reversals and determined that the columns reinforced with high-strength longitudinal reinforcements presented a similar flexural bearing capacity and deformability, but lower energy-dissipating capacity when compared with columns reinforced by conventional steel. Similar results on the seismic behavior of slender columns reinforced with high-strength longitudinal reinforcements can also be found in the literature [14,20,21]. Ou et al. [11,12] and Sokoli et al. [13] studied the shear behavior of large-scale concrete columns reinforced by high-strength steel and concluded that bond degradation around the longitudinal reinforcement occurred and the shear strength of columns was affected by axial compression. This suggests that the minimum shear reinforcement equation needs to consider the influence of axial compression.

The available literature related to the use of new developed high-strength steel mainly focuses on the seismic behavior of concrete beams, columns, beam–column joints, and walls, while studies on the eccentric compressive behavior of columns reinforced with new developed high-strength steel are scare. This study investigated the behavior of new developed high-strength steel used in concrete columns under eccentric loading. Ten large-scale concrete square columns with high-strength steel were designed to explore the effect of using new developed high-strength steel. In addition, this paper compares the different analytical models used to predict the axial load-bending moment interaction curves of high-strength steel reinforced square concrete columns.

## 2. Experimental Program

### 2.1. Test Specimens

**Figure 2:**
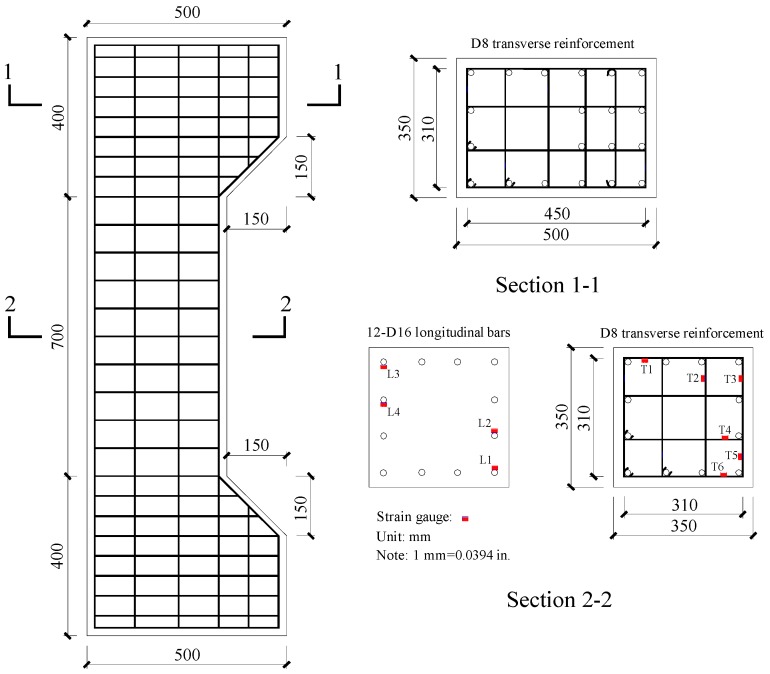
Geometric sizes and reinforcement details.

Ten concrete columns were designed and fabricated with a square section (350 × 350 mm) and a column height of 1500 mm. The test region of columns with height of 700 mm and two column ends (each 400 mm in height) were haunched. The concrete cover thickness of the columns was 20 mm. The test regions of columns were constructed using 12 longitudinal reinforcements with a diameter of 16 mm, and the percentage of longitudinal reinforcements (*ρ_l_*) was 1.97%. The columns were confined by well-shaped compound hoops with 135° bend anchorages. The transverse reinforcement spacing in the test region was 70 mm or 105 mm, and the corresponding transverse reinforcement ratios (*ρ_sh_*) were 1.91% and 1.28%, respectively. The two haunched heads were fabricated with dense transverse reinforcements of 50 mm to prevent local compression failure at the column ends. The geometric sizes and reinforcing bars of the columns are illustrated in Figure 2. Table 1 presents the test parameters in detail.

### 2.2. Test Variables

This test aims to study the three main variables that affect the eccentric compressive behavior of concrete columns: (1) the effect of using high-strength transverse reinforcement and its spacing; (2) the effect of using high-strength longitudinal reinforcements; (3) the effect of eccentricity. Clause 18.7.5.3 in America Code ACI 318-14 [22] specifies the limits for transverse reinforcement spacing. The transverse reinforcement spacing should not exceed: (1) a quarter of the minimum member cross-sectional dimension; (2) six times the longitudinal bar diameter; and (3) *s_o_* as defined by Eq. (18.7.5.3). These limitations lead to a maximum transverse reinforcement spacing of 87.5 mm for the columns. Four specimens, EC3, EC5, EC8, and EC10, do not meet the ACI 318-14 requirement on maximum transverse reinforcement spacing. All columns were separated into two groups. The first group consisted of five concrete columns (EC1, EC2, EC3, EC4, and EC5). Columns from the first group were loaded under a nominal eccentricity of 80 mm (*e*/*h* = 0.23). In the second group, a total of five concrete columns (EC6, EC7, EC8, EC9, and EC10) were examined under a nominal eccentricity of 180 mm (*e*/*h* = 0.51). The detailed test variables are presented in Table 1.

Columns EC2 and EC6 were reinforced with D8 high-strength transverse reinforcements and had the same transverse reinforcement configuration as columns EC1 and EC5, which were reinforced by ordinary transverse reinforcements, to investigate the effects of transverse reinforcement yield strength. The ratio of the configured high-strength stirrup amounted to the amount specified by ACI 318-14 [22], which was 1.72; whereas the ratio of the configured ordinary stirrup amounted to the amount required by ACI 318-14, which was 1.27. Columns EC3 and EC7, which were confined by high-strength stirrups but with a reduced amount compared with the previous four columns, were designed to understand the effects of the high-strength transverse reinforcement amount.

Columns EC4, EC5, EC9, and EC10 were confined by the same transverse reinforcement configuration as columns EC2, EC3, EC7, and EC8, respectively, but differed in longitudinal reinforcement yield strength. Columns EC4, EC5, EC9, and EC10 were fabricated with high-strength longitudinal reinforcements as to be compared with columns EC2, EC3, EC7, and EC8 which were reinforced with ordinary longitudinal reinforcements. The comparison of the behavior of these columns aims to understand the influences of high-strength longitudinal reinforcement.

### 2.3. Material Properties

All columns were cast using the same batch of concrete mix. The materials involved were Portland cement, river sand, coarse aggregate, tap water for mixing and curing, fly ash, and mineral powder to improve the workability of the material. The ratio of sand to coarse aggregate was 0.36 and the water–bind ratio was 0.32. A small amount of superplasticizer was mixed in to increase concrete fluidity. Six plain concrete cubes with a width of 150 mm were fabricated and tested on the day that the columns were loaded to obtain average concrete compressive strength *f*_c_’ according to the China Code GB 50010-2010 Code, as shown in Table 1.

D16 HRB400 and D16 HRB600 reinforcing bars served as longitudinal reinforcements. The HRB400 and newly developed HRB600 represent the minimum specified yield strength of 400 MPa and 600 MPa, respectively. All columns were confined by D8 HRB400 or HRB600 transverse reinforcements. Tensile tests of the steel samples were conducted to obtain the properties of each type of reinforcing bar. The details of the reinforcing bars are given in Table 2.

### 2.4. Test Setup and Loading

**Figure 3:**
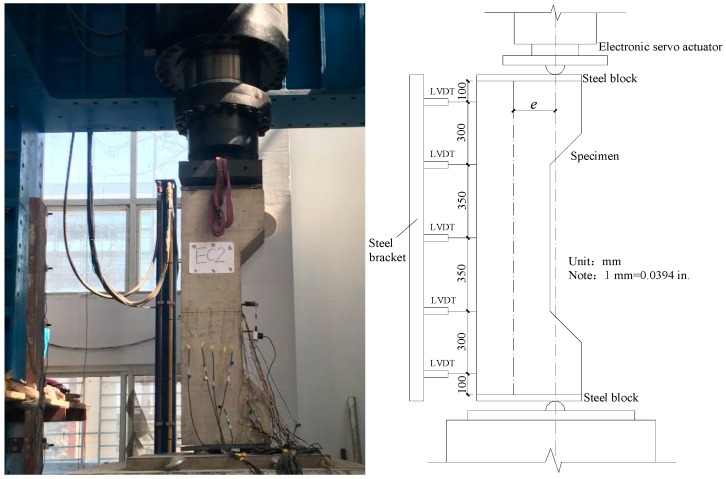
Typical test setup and instrumentation.

All columns were subjected to eccentric loading using a 10,000 kN capacity electro-hydraulic servo machine (Sanke Instrument Company, Chongqing, China), as shown in Figure 3. The top ends of the columns were connected with an electro-hydraulic actuator and the lower ends were placed on a steel block. Both ends of the columns were designed as a hinge connection. The columns were loaded with 0.02 mm/s until the end of the test. Five linear voltage differential transducers (LVDTs) (Hongmei Technology Company, Shenzhen, China) were installed along the height of the columns to measure the lateral deformation. Two of the LVDTs were fixed near the column ends, and an LVDT was installed at the mid-height of the column to obtain the maximum flexural deflection. The remaining two LVDTs were placed at approximately one quarter and three quarters of the column height, respectively. The detailed instrumentations are illustrated in Figure 3. Strain foil was used to measure the strain in the reinforcing bars. The strain foil of longitudinal reinforcements and transverse reinforcements were installed at the test region; detailed locations are illustrated in Figure 2.

## 3. Experimental Results

### 3.1. Failure Patterns

**Figure 4:**
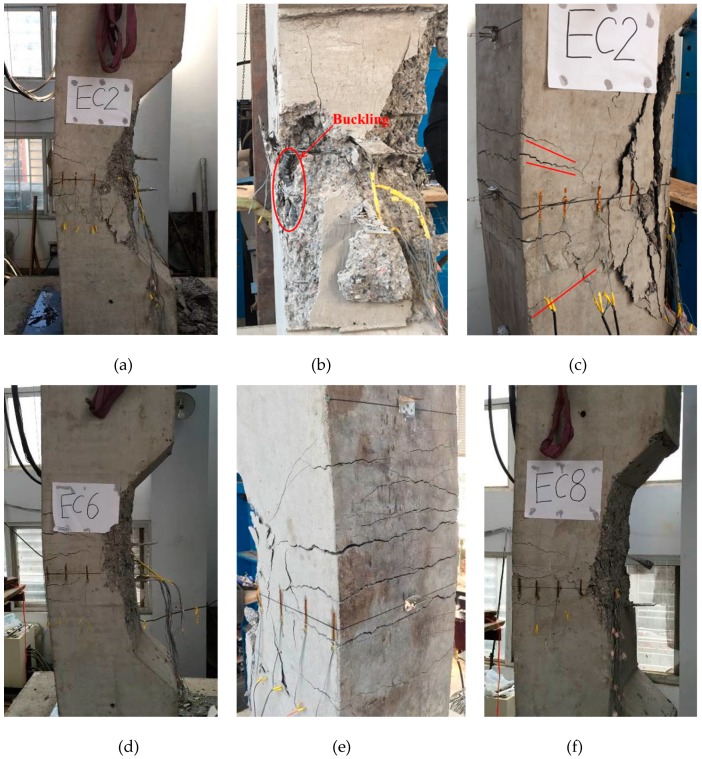
Failure patterns of the columns. (**a**) Final failure mode; (**b**) buckling of longitudinal bar; (**c**) tensile cracks; (**d**) final failure mode of EC6; (**e**) major cracks; (**f**) final failure mode of EC8.

The columns subjected to a small-eccentric compressive loading (nominal *e* = 80 mm) failed at the mid-height, as demonstrated in Figure 4a. The failure patterns of the five columns were similar; compression failure occurred despite the high-strength steel used in the columns. At the beginning of loading, most of the cross-section regions of the columns were compressive. As the displacement increased, the concrete on the compression side crushed suddenly, followed by a buckling outward of the longitudinal reinforcement (Figure 4b). The failure of the columns was determined by concrete compression crushing on the compression side. Under the small eccentric compression loading, tensile cracks were observed, though they did not extend through the entire cross-section of the column (Figure 4c). This is because the confinement effects caused by the transverse reinforcement limited the development of cracks, which produced the core concrete to be under triaxial compression status. Thus, the failure pattern of the columns with small eccentricity was concrete crushing on the compression side.

The columns subjected to large-eccentric compressive loading (nominal *e* = 180 mm) also failed at the mid-height of the columns, as given in Figure 4d. The breakings patterns of the five columns were almost identical, with typical tensile failure patterns, which were caused by the large bending moment at the mid-height of the columns. The bending moment at the mid-height started the cracking of the concrete. As the load increased, the tensile cracks propagated from the outside of the cross-section to the inside. Finally, the tensile cracks extended to the entire cross-section of the columns, with several major cracks in the tensile zones (Figure 4e) and concrete crushed on the compression side. The longitudinal reinforcements on the compression side buckled and the concrete in the middle of the cross-section cracked and peeled off slowly, which can be observed in Figure 4f. For the columns with different reinforcing bar amounts and yield strengths, the failure patterns and post-peak deformability of the columns did not change significantly.

### 3.2. Load-Displacement Behavior

**Figure 5:**
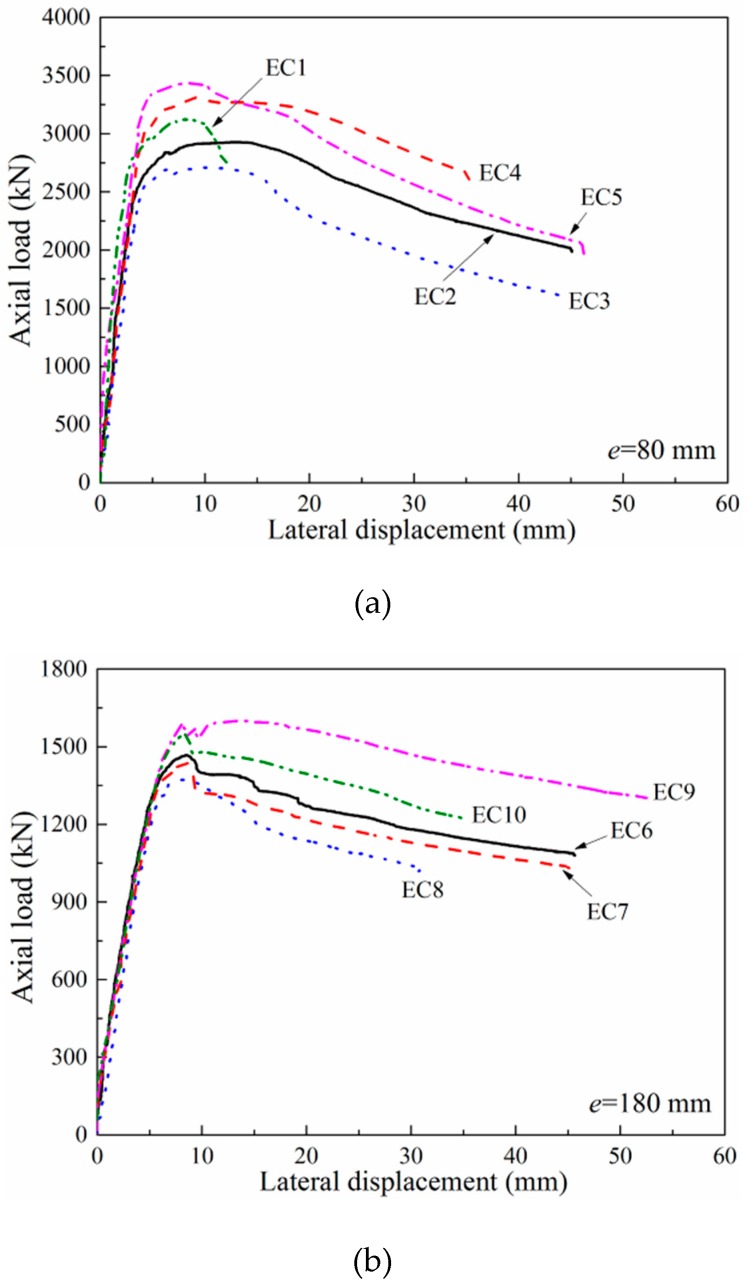
Load-displacement curves of the columns (**a**) nominal eccentricity (*e* = 80 mm); (**b**) nominal eccentricity (*e* = 180 mm).

The lateral displacements versus the applied load curves for the columns are illustrated in Figure 5. The lateral displacement was obtained from LVDTs placed at the mid-height. All columns have an approximate linear load-displacement curve up to the yield point, when the longitudinal reinforcement yielded; the lateral displacement was approximately 4 mm for columns loaded to small eccentricity (*e* = 80 mm), and 5.5 mm for columns subjected to large eccentricity (*e* = 180 mm). After the yield point, the plastic hinge was generated near the mid-height and the stiffness of columns decreased. This behavior continued until the concrete cover underwent crushing and spalling, which caused a sudden decrease of approximately 5% to 10% of the maximum load. The average lateral displacement was 9.0 mm when the first peak load was reached. The applied load increased again until a second peak appeared for the strongly confined columns only (e.g., EC1, EC2, and EC9), and the corresponding lateral displacement ranged from 13.1 mm to 13.95 mm. For columns loaded to small eccentricity (*e* = 80 mm), the post-peak deformability was less than that of the columns subjected to large eccentricity (*e* = 180 mm), and the post-peak behavior was similar to that of the columns under the concentric compression loading. The transverse reinforcements provided a lateral confinement effect to the core concrete, and increased the post-yield deformation capacity of the columns. Therefore, the transverse reinforcement amounts, and yield strength had a significant effect on the confinement effect of concrete and dominated the post-peak behavior of the columns. For the columns loaded to large eccentricity (*e* = 180 mm), the post-peak behavior was similar to that of the flexural members, and they failed in ductility behavior. The compression zones of the critical cross-section for the columns were relatively small and the transverse reinforcements did not develop lateral confining pressure. Therefore, the effects of the transverse reinforcement amounts and yield strength on the behavior of the columns were minor.

### 3.3. Lateral Deformation

**Figure 6:**
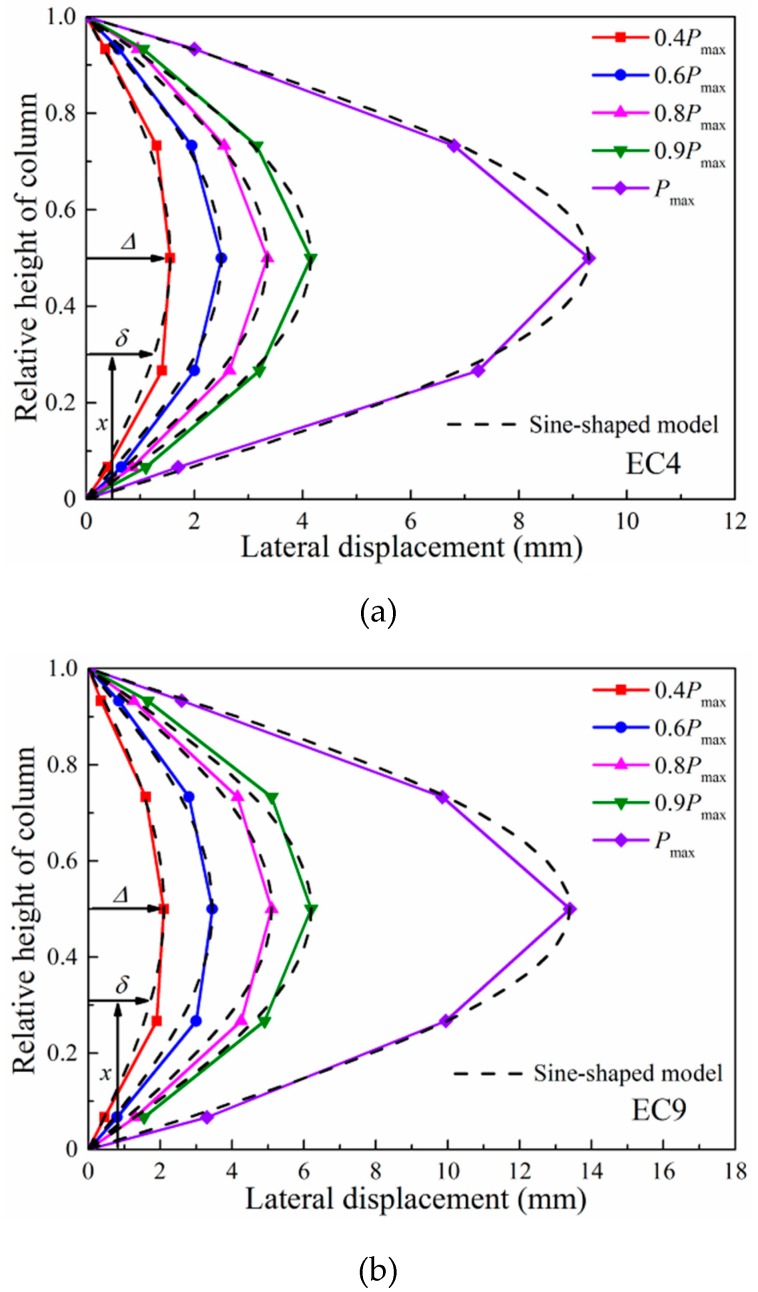
Lateral deformation of columns: (**a**) EC4 (*e* = 79 mm); (**b**) EC9 (*e* = 179 mm).

The lateral deformations recorded by the five LVDTs are illustrated in Figure 6. The lateral deformation of the columns presented a similar shape at the different applied loadings. Particularly, the lateral displacements of the columns increased significantly when the applied load increased to the maximum load. The lateral displacements of the columns subjected to large eccentricity were larger than those of the columns subjected to small eccentricity at different loading stages. The lateral deformation of the columns was caused by the bending moment and is often assumed to be sine-shaped [23,24]; the expression is as follows:

(1) $$\delta =\Delta \sin(\frac{\pi x}{L})$$

where Δ is the lateral displacement at the mid-height of the column; *x* is the longitudinal coordinate variable, as shown in Figure 6; *L* is the height of column; and *δ* is the lateral displacement at the *x* position, as given in Figure 6.

A comparison of the sine-shaped model with experimental lateral deformation is shown in Figure 6. It indicates that the sine-shaped model agrees well with the experimental data for the column cross-section of 350 mm × 350 mm. This model provides a good prediction for the lateral deformation of the columns at different levels of deformation. Thus, the sine-shaped model can be applied to reinforced concrete columns with high-strength steel.

### 3.4. Measured Strains in Longitudinal and Transverse Reinforcement

Table 3 summarizes the load at first yielding of longitudinal reinforcement and steel strains at the maximum load (*P_max_*). The first longitudinal reinforcement yielding appeared on the compression side of the columns subjected to small eccentricity (*e* = 80 mm), while it happened on the tension side of the columns subjected to large eccentricity (*e* = 180 mm). The steel yielding appeared at 0.779 *P_max_* on average, ranging from 0.712 to 0.831 *P_max_*, for the columns reinforced with ordinary longitudinal reinforcements and at 0.904 *P_max_* on average, ranging from 0.845 to 0.938 *P_max_*, for the columns with high-strength longitudinal reinforcements. At maximum load, the average ratios (*ε_lc,Pmax_*/*ε_y_*) of the measured strain to yield strain in compressive longitudinal reinforcements for the columns ranged from 4.00 to 10.95, which indicate that the compressive longitudinal reinforcements could yield despite the use of high-strength longitudinal reinforcements. However, the average ratios (*ε_lt,Pmax_*/*ε_y_*) of the measured strain to yield strain in the tensile longitudinal reinforcements ranged from 0.67 to 1.86 for the columns subjected to small eccentricity and ranged from 1.50 to 5.24 for the columns subjected to large eccentricity, which indicate that the tensile high-strength longitudinal reinforcements could not yield (*ε_lt,Pmax_*/*ε_y_* = 0.67/0.90) for the columns loaded to small eccentricity. The average strains (*ε_hc,Pmax_*) of the transverse reinforcement on the compression side ranged from 1.03 to 2.03 of yield strain of the transverse reinforcements for columns subjected to small eccentricity, which is similar to the behavior of the columns subjected to concentric compression loading. It indicates that transverse reinforcements have an obvious confinement effect on the concrete. However, the average strain (*ε_hc,Pmax_*) of the transverse reinforcements on the compression side ranged from 0.39 to 0.79 of yield strain for the columns loaded to large eccentricity, which shows that the confinement effect of the transverse reinforcement on the core concrete was not significant due to the low stress of the transverse reinforcements. The average strains (*ε_ht,Pmax_*) of the transverse reinforcements on the tension side ranged from 0.02 to 0.39 of yield strain of transverse reinforcement for all the columns, which is much less than the yield strain. This is due to the stress release of the transverse reinforcements caused by the cracking of the concrete on the tension side.

### 3.5. Summary of Test Results

Table 4 summarizes the test results, including the maximum load *P_max_* and corresponding mid-height lateral displacement Δ*_max_*, calculated maximum loads *P*_ACI_, *P*_GB_ and *P*_CSA_ using the ACI 318-14 [22], GB 50010-2010 [25] and Canada Code CSA A23.3-04 [26] Codes, respectively, the yield displacement Δ*_y_* and the displacements Δ*_0.85P_* corresponding to 0.85 of the maximum load. The maximum loads of the columns were calculated using the equivalent rectangular stress block (ERSB) parameters stated in ACI 318-14, GB 50010-2010, and CSA A23.3-04. There are differences in the calculation of maximum loads in the above three codes. ACI 318-14 defines ERSB width and depth using parameters α and β, respectively. Factor α is given as 0.85. The concrete ultimate compression strain is taken as *ε*_cu_ = 0.003. Factor *β* defined by ACI 318-14 is given as follows:

(2) $$\beta =0.85\beta =0.85\left(f_{c}^{’}\leq 30\mathrm{MPa}\right)$$

(3) $$\beta =0.85-0.05\frac{\left(f_{c}^{’}-30\right)}{7}\geq 0.65\left(f_{c}^{’}\geq 30\mathrm{MPa}\right)$$

The ERSB in the China Code (GB 50010-2010) is explained using the *α* and *β* parameters. Factor *α* and *β* should be taken as 1.0 and 0.80 for concrete strength grade up to and including C50 (cube concrete strength *f_cu_*’ = 50 MPa), respectively. For the concrete strength grade of C80 (cube concrete strength *f_cu_*’ = 80 MPa), factor *α* and *β* can be taken as 0.94 and 0.74, respectively. While for the concrete strength grade between C50 and C80, parameters *α* and *β* should be determined using the linear interpolation method. The concrete ultimate compression strain is taken as *ε*_cu_ = 0.0033.

The Canada CSA A23.3-04 Code defines the ERSB, using factors *α* and *β* as follows:

(4) $$\alpha =0.85-0.0015f_{c}^{’}\geq 0.67$$

(5) $$\beta =0.97-0.0025f_{c}^{’}\geq 0.67$$

The specified concrete compressive strength ranges from 20 MPa to 80 MPa. The ultimate compression strain is taken as *ε*_cu_ = 0.0035.

As shown in Table 4, the ratio of *P_max_* to *P*_ACI_ was 1.03 on average, ranging from 0.91 to 1.15, the ratio of *P_max_* to *P*_GB_ was 0.91 on average, ranging from 0.83 to 0.99, and the ratio of *P_max_* to *P*_CSA_ was 1.04 on average, ranging from 0.93 to 1.15. The theoretical loads of the columns obtained according to GB 50010-2010 Code were larger than the experimental maximum loads. The theoretical loads calculated using ACI 318-14 and CSA A23.3-04 Codes were close to the experimental results, and the standard deviations of mean ratios were 0.074 and 0.071, respectively. The average ratio of experimental maximum load to theoretical load obtained using codes decreased with the increase of nominal eccentricity. In the theoretical calculations of the columns with high-strength steel, the ERSB parameters given in ACI 318-14 and CSA A23.3-04 Codes were more suitable than the GB 50010-2010 Code.

**Figure 7:**
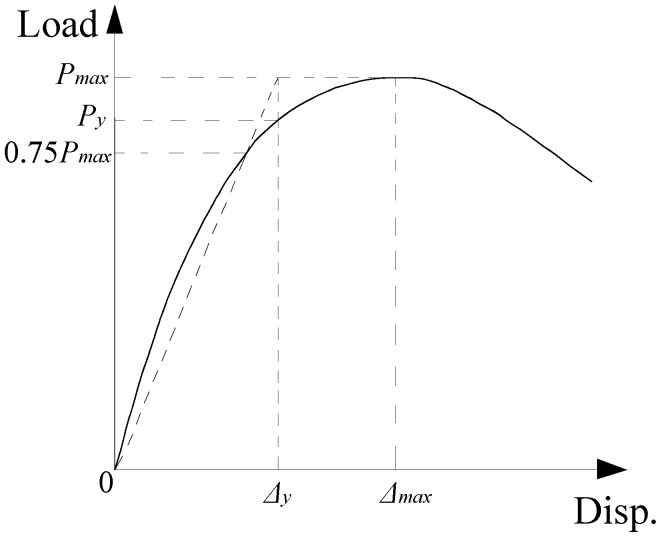
Yield point determined by the Park et al. method.

To evaluate the effects of test design parameters on the post-peak deformability and ductility of the columns, the deformability factor *λ* [27] and ductility index *μ* [28] were used and are presented in Table 4. The deformability factor *λ* is defined as the ratio of mid-height lateral displacement at ultimate load to mid-height lateral displacement at peak load. The ultimate load was determined by the load corresponding to 85% of the maximum load on the descending branch [29]. The ductility index *μ* is defined as the ratio of ultimate lateral displacement to notional yield displacement. The yield displacement was given as the displacement at the yield point of load-displacement curves, which can be determined by the method proposed by Park et al. (as shown in Figure 7).

## 4. Discussion of Test Results

### 4.1. Effect of Transverse Reinforcement Amount

According to the load-displacement curves of the columns illustrated in Figure 5 and the test results shown in Table 4, four pairs of columns (EC2 and EC3, EC4 and EC5, EC7and EC8, EC9 and EC10) containing high-strength transverse reinforcement were presented, with each pair having the same transverse reinforcement configurations but in different amounts. Column EC2 exhibited 8%, 5%, and 44% increases in maximum load *P_max_*, deformability *λ*, and ductility *μ*, respectively, compared with column EC3. Similarly, column EC4 presented a 4% decrease in maximum load *P_max_*, but 27% and 23% gains in deformability *λ* and ductility *μ*, respectively, compared with column EC5. For the columns subjected to a nominal eccentricity of 180 mm, column EC7 increased by 5%, 15%, and 13% in maximum load *P_max_*, deformability *λ* and ductility *μ*, respectively, compared with column EC8. Column EC9 presented a 3% and 57% increase in maximum load *P_max_* and ductility *μ*, respectively, compared with column EC10. These results demonstrate the beneficial effect of transverse reinforcement amount on the behavior of the columns under eccentric loading on the basis of deformability and ductility and also show that the confinement effect of the transverse reinforcements is more important in columns loaded to small eccentricity (*e* = 80 mm) than columns subjected to large eccentricity (*e* = 180 mm). This is due to the lower amount of core concrete confined by transverse reinforcement for columns subjected to large eccentricity, which results in the low effect of confinement. 

### 4.2. Effect of Transverse Reinforcement Yield Strength

The effects of transverse reinforcement yield strength on the eccentric compressive behavior of the columns are shown in Figure 5 and Table 4. Column EC2 with 1.91% of high-strength transverse reinforcement exhibited 33% and 71% increases in deformability *λ* and ductility *μ*, respectively, compared with column EC1, which had 1.91% of the ordinary transverse reinforcement. These results indicate that increasing transverse reinforcement yield strength has beneficial effects on the post-peak deformability and ductility of columns subjected to small eccentricity. However, column EC7 with 1.91% of the high-strength transverse reinforcements showed 15% and 19% decreases in deformability *λ* and ductility *μ*, respectively, compared with column EC6 with 1.91% of the ordinary transverse reinforcements. This is because the transverse reinforcements cannot yield despite the use of high-strength transverse reinforcement in columns (refer to measured strains in Table 3). In addition, columns EC3 and EC8 were detailed with high-strength transverse reinforcements but have a smaller transverse reinforcement amount (*ρ_sh_* = 1.27%) to columns EC1 and EC6 (*ρ_sh_* = 1.91%), respectively. Columns EC3 and EC8 exhibited 14% and 7% decreases in maximum load *P_max_* compared with columns EC1 and EC6, respectively. This is attributed to the smaller mechanical ratio (*ρ_sh_f_yh_*/*f_c_*’) proposed by Canbay et al. [30]. Column EC3 showed 26% and 19% increases in deformability *λ* and ductility *μ*, respectively, compared with EC1, although it contained a lower transverse reinforcement amount. However, column EC8 exhibited 26% and 29% decreases in deformability *λ* and ductility *μ*, respectively, compared with column EC6. These results show that the same level of deformability and ductility can only be achieved with lower amounts of transverse reinforcements when high-strength transverse reinforcements are used in columns subjected to small eccentricity. That is, the beneficial effects of using high-strength transverse reinforcements to solve steel congestion in the columns can be achieved for columns subjected to small eccentricity. Therefore, the confinement effect of high-strength transverse reinforcements was not effective in columns subjected to large eccentricity. 

### 4.3. Effect of Longitudinal Reinforcement Yield Strength

The influence of longitudinal reinforcement yield strength on the behavior of the columns is illustrated in Figure 5 and Table 4. Columns EC4, EC5, EC9, and EC10 with high-strength longitudinal reinforcements exhibited 13%, 27%, 11%, and 13% increases in maximum load *P_max_* compared with columns EC2, EC3, EC7, and EC8, respectively. These results demonstrate that the high-strength longitudinal reinforcements can increase the bearing capacity of the columns. That is, the beneficial effects of using high-strength longitudinal reinforcements to solve steel congestion in columns with large amounts of reinforcement can be achieved. Columns EC4 and EC5 exhibited similar ductility *μ* (a 3% decrease and 9% increase, respectively) compared with columns EC2 and EC3, respectively. While columns EC9 and EC10 increased by 103% and 47% in ductility *μ* compared with columns EC7 and EC8, respectively. These results indicate that the benefits of using high-strength longitudinal reinforcements for improving ductility can be achieved in columns subjected to large eccentricity. In addition, columns EC4, EC5, EC9, and EC10 showed 65%, 37%, 39%, and 65% increases in deformability *λ* compared with columns EC2, EC3, EC7, and EC8, respectively, which indicates that the high-strength longitudinal reinforcements can clearly improve post-peak deformability of the columns.

## 5. Axial Load-Bending Moment Interaction Diagrams

### 5.1. Bending Moment M

The behavior of the columns with varying design parameters cannot be fully covered experimentally. Hence, numerical models were established using Software OpenSees to extensively investigate the effect of concrete strength *f*_c_’, eccentricity *e*, and slenderness ratio *λ* on the bending moment of the columns with high-strength steel. These included investigating concrete strengths of *f*_c_’ = 20 MPa, 40 MPa, and 50 MPa under eccentricities of *e* = 80 mm, 160 mm, 240mm; eccentricities of *e* = 40 mm, 80 mm, 120 mm, 160 mm, 200 mm, 240 mm, 280 mm, and 320 mm under concrete strength *f*_c_’ = 30 MPa; slenderness ratios *λ* = 6, 9, 12, and 15 under eccentricities *e* = 80 mm, and 240mm. The finite element model is based on nonlinear beam-column elements with fiber sections. Herein, the fiber section includes 52 fibers for the unconfined cover, 144 fibers for the confined core, and one fiber for each longitudinal reinforcing bar. Concrete 02 and reinforcing steel material models in Opensees were used to simulate the concrete and steel constitutive of the columns, respectively. The Concrete 02 takes into account the tensile properties of the concrete and the stiffness degradation in unloading compared with other concrete constitutive models. Furthermore, the test results in the literature [31] were also collected for comparison. The bending moment capacities of the columns were calculated using the ERSB parameters stated in the ACI 318-14, GB 50010-2010, and CSA A23.3-04 Codes. The experimental, simulated, and calculated bending moment capacities are summarized in Table 5.

As a result of the nature of eccentric loading, the axial load produced a large bending moment at the mid-height of the column. The bending moment capacity *M* of the columns reported in Table 5 consists of the primary moment calculated based on nominal eccentricity and the secondary moment caused by lateral mid-height displacement at maximum load (*P*-Δ effect). The actual *e*/*h* for each series of columns was approximately equal and hence any change in bending moment capacity was due to the test variables. As shown in Table 5, the bending moment capacity caused by the test variables decreased as *e*/*h* increased. The average ratios of the experimental and simulation bending moment capacity (*M*) to bending moment capacity (*M*_ACI_) computed using the ACI 318-14 Code, *M*/*M*_ACI_ was 1.10; the bending moment capacity (*M*_GB_) according to GB 50010-2010 Code, *M*/*M*_GB_ was 0.98; and the bending moment capacity (*M*_CSA_) calculated according to the CSA A23.3-04 Code, *M*/*M*_CSA_ was 1.07. The bending moment capacity calculated according to the ACI 318-14 and CSA A23.3 Codes were less than those of the experimental and simulation results, which indicates that the calculated results are conservative. While the bending moment capacities calculated by GB 50010-2010 Code were bigger than the experimental and simulation results, which shows that the GB 50010-2010 Code overestimates the bending moment capacity.

### 5.2. P–M Curve Diagram

**Figure 8:**
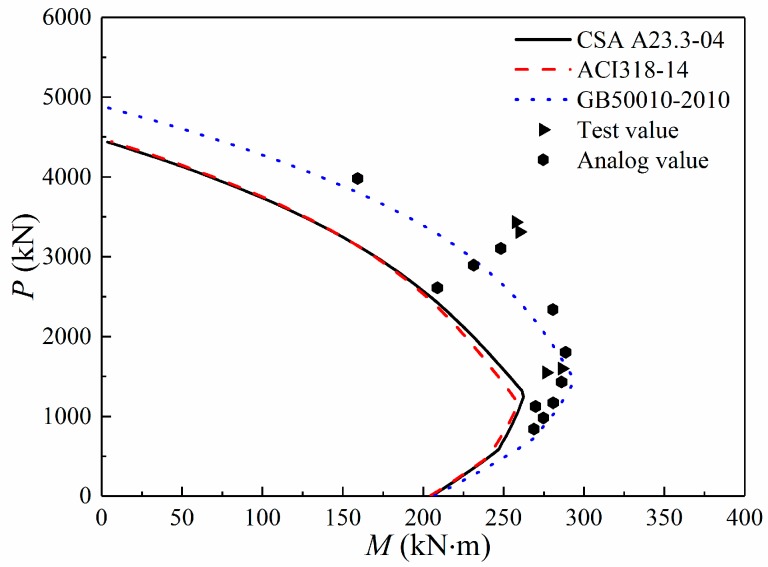
*P–M* curve diagrams of the columns with high-strength steel.

According to the detailed parameters of the columns given in the test, the axial load *P* and bending moment *M* of the columns can be calculated in terms of the relevant formulas from the GB50010-2010, ACI318-14, and CSA A23.3-04 Codes. For different eccentricities, the axial load-bending moment (*P–M*) curves are shown in Figure 8. The *P–M* interaction curves calculated using the GB 50010-2010 Code give an upper bound, and the *P–M* interaction curves obtained using the ACI 318-14 Code almost coincide with the CSA A23.3-04 curves. The results remain on the unsafe side of the *P–M* curve obtained using the GB 50010-2010 Code, whereas the experimental and simulation points are close to those of the ACI 318-14 and CSA A23.3-04 curves. The ACI 318-14 and CSA A23.3-04 Codes give the safest predictions. The GB 50010-2010 Code overestimates the capacity for the columns with high-strength steel. In general, capacities calculated using the ACI 318-14 and CSA A23.3-04 Codes agree well with the test results.

## 6. Conclusions

This study aimed to investigate the eccentric compressive behavior of RC columns with new developed high-strength steel. The transverse reinforcement amount and yield strength, eccentricity, and longitudinal bar yield strength were the test variables. In total, 10 concrete columns were tested under eccentric loading. According to the experimental and analytical results, some conclusions were obtained:

1. For small-eccentrically loaded concrete columns reinforced with high-strength steel, the failure was located in the middle part with concrete crushing on the compression side and compression failure occurring. For large-eccentrically loaded concrete columns with high-strength steel, the tensile cracks extended through the entire cross-section of the columns with several major cracks in the tensile zones and concrete crushed on the compression side, which is a typical tensile failure mode.
2. Increasing the amount of transverse reinforcements improved the deformability and ductility of the columns with high-strength steel. In addition, the confinement effect of transverse reinforcements was more effective in columns subjected to small eccentricity than columns subjected to large eccentricity.
3. The same level of deformability and ductility could be achieved with a lower amount of transverse reinforcement when high-strength transverse reinforcements were used in columns subjected to small eccentricity. However, increasing the transverse reinforcement yield strength failed to improve post-peak deformability and ductility of the columns subjected to large eccentricity due to the inadequate confinement of the high-strength transverse reinforcement.
4. High-strength longitudinal reinforcements improved the bearing capacity and post-peak deformability of columns under eccentric loading, but the benefits of using high-strength longitudinal reinforcements for improving ductility can only be achieved in columns subjected to large eccentricity.
5. In general, the equivalent rectangular stress block (ERSB) parameters stated in the GB 50010-2010 Code overestimate the bearing capacity of columns with high-strength steel, whereas the bearing capacities computed using the ACI 318-14 and CSA A23.3-04 Codes better agree with the test results.

## Figures and Tables

**Table 1:** Test design parameters.

Column	Concrete	Eccentricity	Longitudinal Reinforcement			Transverse Reinforcement					
	*f*_c_’ (MPa)	*e* (mm)	No.-Diameter	*f_yl_* (MPa)	*ρ_l_* (%)	Size	Spacing (mm)	*f_yh_* (MPa)	*ρ_sh_* (%)	*ρ_sh_f_yh_*/*f*_c_’	*ρ_sh_*/*ρ_sh(ACI)_*
EC1	36.5	75	12-D16	446	1.97	D8	70	476	1.91	0.296	1.27
EC2	36.5	74	12-D16	446	1.97	D8	70	642	1.91	0.399	1.72
EC3	36.5	80	12-D16	446	1.97	D8	105	642	1.28	0.268	1.15
EC4	36.5	79	12-D16	617	1.97	D8	70	642	1.91	0.399	1.72
EC5	36.5	75	12-D16	617	1.97	D8	105	642	1.28	0.268	1.15
EC6	36.5	177	12-D16	446	1.97	D8	70	476	1.91	0.296	1.27
EC7	36.5	180	12-D16	446	1.97	D8	70	642	1.91	0.399	1.72
EC8	36.5	176	12-D16	446	1.97	D8	105	642	1.28	0.268	1.15
EC9	36.5	179	12-D16	617	1.97	D8	70	642	1.91	0.399	1.72
EC10	36.5	179	12-D16	617	1.97	D8	105	642	1.28	0.268	1.15

**Table 2:** Properties of the reinforcing bars.

	Size	Grade	Elasticity Modulus (MPa)	Yield Strength (MPa)	Yield Strain	Ultimate Strength (MPa)
Longitudinal reinforcement	D16	HRB400	2.08 × 10^5^	446	0.0021	585
	D16	HRB600	2.05 × 10^5^	617	0.0030	802
Transverse reinforcement	D8	HRB400	2.00 × 10^5^	476	0.0024	635
	D8	HRB600	2.04 × 10^5^	642	0.0031	803

**Table 3:** Measured strains in longitudinal and transverse reinforcement.

Column	*P_max_* (kN)	Load at First Yielding of Longitudinal Bar		Average Strains in Longitudinal Bar				Average Strains in Transverse Reinforcement			
		*P_yl_* (kN)	*P_yl_*/*P_max_*	*ε_lt,Pmax_*	*ε_lt,Pmax_*/*ε_y_*	*ε_lc,Pmax_*	*ε_lc,Pmax_*/*ε_y_*	*ε_ht,Pmax_*	*ε_ht,Pmax_*/*ε_y_*	*ε_hc,Pmax_*	*ε_hc,Pmax_*/*ε_y_*
EC1	3125	2423	0.775	0.0030	1.43	0.014	6.67	0.0002	0.083	0.0031	1.29
EC2	2927	2380	0.813	0.0039	1.86	0.014	6.67	0.0012	0.387	0.0063	2.03
EC3	2709	2250	0.831	0.0028	1.33	0.015	7.14	0.0003	0.097	0.0036	1.16
EC4	3311	2798	0.845	0.0020	0.67	0.015	5.00	0.0009	0.290	0.0032	1.03
EC5	3434	3223	0.938	0.0027	0.90	0.014	4.67	0.0004	0.129	0.0034	1.10
EC6	1467	1166	0.790	0.0110	5.24	0.020	9.52	0.0004	0.167	0.0019	0.79
EC7	1437	1023	0.712	0.0087	4.14	0.019	9.05	0.0003	0.097	0.0014	0.45
EC8	1373	1031	0.751	0.0085	4.05	0.023	10.95	0.0003	0.097	0.0015	0.48
EC9	1599	1452	0.908	0.0045	1.50	0.017	5.67	0.0006	0.019	0.0022	0.71
EC10	1549	1429	0.923	0.0045	1.50	0.012	4.00	0.0004	0.129	0.0012	0.39

**Table 4:** Summary of test results.

Column	Axial loads				Mid-Height Lateral Displacements			Deformability	Ductility
	*P_max_* (kN)	*P_max_*/*P*_ACI_	*P_max_*/*P*_GB_	*P_max_*/*P*_CSA_	Δ*_y_* (mm)	Δ*_max_* (mm)	Δ*_0.85P_* (mm)	*λ*	*μ*
EC1	3125	1.08	0.95	1.12	2.89	8.30	12.05	1.5	4.2
EC2	2927	1.01	0.88	1.04	3.60	13.10	26.45	2.0	7.2
EC3	2709	0.97	0.85	1.00	3.95	10.25	19.85	1.9	5.0
EC4	3311	1.14	0.98	1.14	4.38	9.30	30.45	3.3	7.0
EC5	3434	1.15	0.99	1.15	3.87	8.55	21.90	2.6	5.7
EC6	1467	0.98	0.89	1.00	5.45	8.60	22.80	2.7	4.2
EC7	1437	0.98	0.89	1.00	5.74	8.45	19.60	2.3	3.4
EC8	1373	0.91	0.83	0.93	5.68	8.55	17.30	2.0	3.0
EC9	1599	1.05	0.91	1.01	6.44	13.95	44.20	3.2	6.9
EC10	1549	1.01	0.88	0.97	6.25	8.30	27.35	3.3	4.4

**Table 5:** Experimental and theoretical bending moment capacity of the columns.

Source	Column			*e* (mm)	*M* (kN·m)	*M*_GB_ (kN·m)	*M*_ACI_ (kN·m)	*M*_CSA_ (kN·m)	*M*/*M*_GB_	*M*/*M*_ACI_	*M*/*M*_CSA_
Test results	EC4			78.5	290.7	274.3	241.1	241.9	1.06	1.21	1.20
	EC5			75	287.0	271.3	237.0	237.1	1.06	1.21	1.21
	EC9			179	308.5	306	277.1	291.3	1.01	1.11	1.06
	EC10			178.5	289.4	306	277.1	291.3	0.95	1.04	0.99
Simulation results	*f*_c_’ (MPa)		20	80	242.2	212.6	177.5	183.0	1.14	1.36	1.32
			40	160	316.3	322.6	307.2	317.1	0.98	1.03	1.00
			50	240	351.3	363.9	389.4	400.5	0.97	0.90	0.88
			20	80	270.2	240.7	212.3	224.6	1.12	1.27	1.20
			40	160	337.5	347.1	325.7	361.1	0.97	1.04	0.93
			50	240	373.1	381.8	393.6	426.3	0.98	0.95	0.88
			20	80	270.0	252.2	224.8	240.4	1.07	1.20	1.12
			40	160	318.2	327.6	318.3	333.8	0.97	1.00	0.95
			50	240	343.5	349.2	360.1	375.0	0.98	0.95	0.92
	*e* (mm)			40	188.1	199.8	172.9	168.6	0.94	1.09	1.12
				80	284.9	275	242.1	243.0	1.04	1.18	1.17
				120	304.6	293.6	264.9	272.6	1.04	1.15	1.12
				160	310.6	302.9	274.2	286.8	1.03	1.13	1.08
				200	304.5	308.3	279.6	292.7	0.99	1.09	1.04
				240	293.8	299.5	281.0	285.1	0.98	1.05	1.03
				280	285.1	292.5	272.9	278.4	0.97	1.04	1.02
				320	277.6	287	266.5	273.1	0.97	1.04	1.02
	*λ*	6		80	286.7	276.8	248.8	250.8	1.04	1.15	1.14
		9		80	282.4	280.9	260.6	265.8	1.01	1.08	1.06
		12		80	292.3	286.7	270.9	281.1	1.02	1.08	1.04
		15		80	287.6	293	277.5	291.9	0.98	1.04	0.99
		6		240	292.0	298.1	279.0	283.5	0.98	1.05	1.03
		9		240	287.7	294.6	274.7	280.1	0.98	1.05	1.03
		12		240	281.1	288.8	269.2	275.6	0.97	1.04	1.02
		15		240	275.3	281.6	264.2	271.4	0.98	1.04	1.01
Literature [31]	PZ1			210	152.7	150.5	150.0	145.1	1.01	1.02	1.05
	PZ2			230	204.9	203.7	192.0	191.6	1.01	1.07	1.07
	PZ3			150	256.2	232.7	209.0	221.5	1.10	1.23	1.16
	PZ4			210	204.1	210.1	189.5	197.3	0.97	1.08	1.03
	PZ5			210	270.3	240.5	217.9	226.8	1.12	1.24	1.19
	PZ6			120	180.5	180.9	165.2	167.8	1.00	1.09	1.08
	PZ7			220	251.6	234.5	215.6	221.2	1.07	1.17	1.14
	PZ8			170	197.5	174.1	172.2	167.9	1.13	1.15	1.18
	PZ9			120	226.6	213.3	193.7	200.0	1.06	1.17	1.13
	PZ3-2			150	264.4	267.9	247.0	259.7	0.99	1.07	1.02
	PZ5-2			210	277.3	237.6	220.2	223.2	1.17	1.26	1.24
Average									0.98	1.10	1.07
